# Transcutaneous auricular vagus nerve stimulation as a potential novel treatment for polycystic ovary syndrome

**DOI:** 10.1038/s41598-023-34746-z

**Published:** 2023-05-12

**Authors:** Shike Zhang, Hui He, Yu Wang, Xiao Wang, Xiaofang Liu

**Affiliations:** 1 Southern University of Science and Technology Yantian Hospital, Shenzhen, 518081 China; 2 Shenzhen Yantian District People’s Hospital, Shenzhen, 518081 China; 3 First Affiliated Hospital of Zhengzhou University, Zhengzhou, 450000 China; 4 First Affiliated Hospital of Heilongjiang University of Chinese Medicine, Harbin, 150040 China; 5 Chinese People’s Liberation Army General Hospital, Beijing, 100853 China

**Keywords:** Endocrinology, Medical research, Neurology

## Abstract

Polycystic ovary syndrome (PCOS) is a common endocrine disorder in women of childbearing age. The etiology of PCOS is multifactorial, and current treatments for PCOS are far from satisfactory. Recently, an imbalanced autonomic nervous system (ANS) with sympathetic hyperactivity and reduced parasympathetic nerve activity (vagal tone) has aroused increasing attention in the pathogenesis of PCOS. In this paper, we review an innovative therapy for the treatment of PCOS and related co-morbidities by targeting parasympathetic modulation based on non-invasive transcutaneous auricular vagal nerve stimulation (ta-VNS). In this work, we present the role of the ANS in the development of PCOS and describe a large number of experimental and clinical reports that support the favorable effects of VNS/ta-VNS in treating a variety of symptoms, including obesity, insulin resistance, type 2 diabetes mellitus, inflammation, microbiome dysregulation, cardiovascular disease, and depression, all of which are also commonly present in PCOS patients. We propose a model focusing on ta-VNS that may treat PCOS by (1) regulating energy metabolism via bidirectional vagal signaling; (2) reversing insulin resistance via its antidiabetic effect; (3) activating anti-inflammatory pathways; (4) restoring homeostasis of the microbiota-gut-brain axis; (5) restoring the sympatho-vagal balance to improve CVD outcomes; (6) and modulating mental disorders. ta-VNS is a safe clinical procedure and it might be a promising new treatment approach for PCOS, or at least a supplementary treatment for current therapeutics.

## Introduction

### The pathological mechanism of polycystic ovary syndrome

Polycystic ovary syndrome (PCOS) is a common reproductive and endocrine disorder with a prevalence of 4–10% in women of childbearing-age depending on the definitions that are used^1^. It’s typical clinical manifestations include menstrual irregularities, clinical and/or biochemical hyperandrogenism (HA), and polycystic ovary morphology on ultrasound^2,3^. Besides reproductive dysfunction, PCOS may result in various metabolic co-morbidities affecting multiple aspects of a woman’s overall health. For example, insulin resistance (IR) is a key characteristic of PCOS, and this can be exacerbated by obesity, particularly visceral adiposity^4^. Women with PCOS also have a markedly increased risk for impaired glucose tolerance^5,6^, type 2 diabetes mellitus (T2DM)^7,8^, and cardiovascular disease (CVD)^9^ owing to the persistent effects of IR on metabolism. In addition, these women are more likely to suffer from psychological illnesses such as anxiety and depression than healthy controls^10–15^.

PCOS is a biologically heterogeneous condition involving multiple pathophysiological processes that lead to ovarian dysfunction. It is considered to be a neuroendocrine disease resulting from an aberrant hypothalamic-hypophyseal system^16,17^. Hypothalamus-pituitary-ovary axis dysregulation—including rapid gonadotrophin-releasing hormone pulse frequency associated with luteinizing hormone (LH) hypersecretion and increased ovarian androgen production—leads to impaired folliculogenesis and oocyte development^18–20^. Adrenally derived androgens due to disruption of the hypothalamic–pituitary–adrenal (HPA) axis may also contribute to the occurrence of PCOS^21^. IR and HA are the most significant hormonal disturbances and are usually regarded as the chief causes of PCOS^22^. In addition, chronic low-grade inflammation may be an important component in the pathophysiology of PCOS and might have additional effects on the long-term metabolic disorders related to PCOS^23,24^. Recently, increasing studies have reported that autonomic dysfunction is also involved in the development of PCOS^25–28^.

### The sympatho-vagal balance

The sympathetic nervous system (SNS) and parasympathetic nervous system (PNS) are two primary branches of the autonomic nervous system (ANS). The vagus nerve (VN), which is the 10th and longest of the cranial nerves with 80%–90% afferent fibers, is a critical constituent of the PNS and acts as an important bidirectional conduit between the body and brain and mainly serves to maintain homeostasis^29,30^. The functions of the SNS and PNS are antagonistic and are in dynamic balance under physiological conditions, and imbalances in the interactions between the SNS and PNS can lead to various autonomic modulation-related disorders, including neurological, metabolic, inflammatory, cardiovascular, and psychiatric diseases^31^. In general, this imbalance involves relatively higher sympathetic activity associated with a paucity of parasympathetic activity^32^.

### Link between ANS dysfunction and PCOS

Ovary function is not only regulated by hormones, but also by neural signals. The ovary is innervated by the sympathetic superior ovarian nerve and the ovarian plexus nerve from the upper lumbar segment via visceral nerve fibers and by the parasympathetic nerve through the VN, which is regulated by the central nervous system (CNS)^33,34^. Abnormalities in the ANS play an important role in the progression of ovarian pathological conditions, such as PCOS. Numerous previous experiments in rats with steroid-induced polycystic ovaries have shown that hyperactivity of the SNS innervating the ovary—which can be demonstrated through enhanced synthesis of SNS activity markers, such as nerve growth factor, norepinephrine, and tyrosine hydroxylase—may contribute to the etiology of PCOS^35–39^. Furthermore, the peripheral SNS may be involved in the pathophysiology of PCOS by modulating immune-endocrine function^40^. In turn, hormonal and metabolic disturbances are also related to autonomic dysfunction in PCOS. Hashim et al. reported that obese women with PCOS exhibited more pronounced autonomic dysfunction and sympathoexcitation than non-obese patients^41^. Shorakae et al. demonstrated that lower high-molecular-weight adiponectin, a biologically active form closely associated with insulin sensitivity and metabolic disorders, is inversely associated with increased sympathetic activity in women with PCOS^42^. Sverrisdottir et al*.* showed that testosterone is positively associated with muscle sympathetic nerve activity in lean women with PCOS^43^, and Shorakae et al. found that chronic low-grade inflammation might play a potential role in mediating the effect of sympathetic dysfunction on HA and IR in PCOS^24^.

Based on the abundant evidence of increased sympathetic neural activity in PCOS documented in both animal and human studies, and the complicated and bidirectional associations of sympathetic activation with endocrinal and metabolic disorders, it is reasonable to speculate that sympathoexcitation may play a role in the progression of the syndrome. Therefore, treatments that seek to reduce sympathetic activity or increase parasympathetic activity in order to restore the sympatho-vagal balance may have the potential to improve the outcomes of PCOS. Indeed, conventional interventions, such as weight loss, pharmacotherapy with the insulin sensitizer metformin, electroacupuncture, etc., have been reported to play a role in suppressing sympathetic over-activation^44^, while parasympathetic (vagal) modulation has been largely neglected.

### Transcutaneous auricular vagus nerve stimulation (ta-VNS)

Vagus nerve stimulation (VNS), a kind of bioelectronic medicine, was first introduced by James Leonard Corning in the late eighteenth century and provided a new way of regulating the autonomic tone^45^. Emerging evidence has verified the improved outcomes of VNS in treating various diseases, and it has been approved by the Food and Drug Administration (FDA) as an alternative therapy for refractory epilepsy, refractory depression, cluster headaches, and migraines^46^. Furthermore, studies have expanded the use of VNS for a wider range of illnesses, including obesity, diabetes, CVD, and chronic inflammatory disorders such as sepsis, lung injury, rheumatoid arthritis, etc.^47^. Initially, VNS was performed by implanting stimulating or surface electrodes into the easily accessible vagus nerve in the neck for acute or chronic stimulation. However, this invasive VNS technique is associated with various surgical complications, for example, infection, cardiac arrhythmia, device malfunction, cough, hoarseness, dyspnea, dysphagia, and so on^48^, thus limiting the application of VNS among larger patient populations. With advancements in medical technology, VNS can now be applied indirectly and non-invasively, including transcutaneous cervical VNS and ta-VNS^49^.

**Figure 1:**
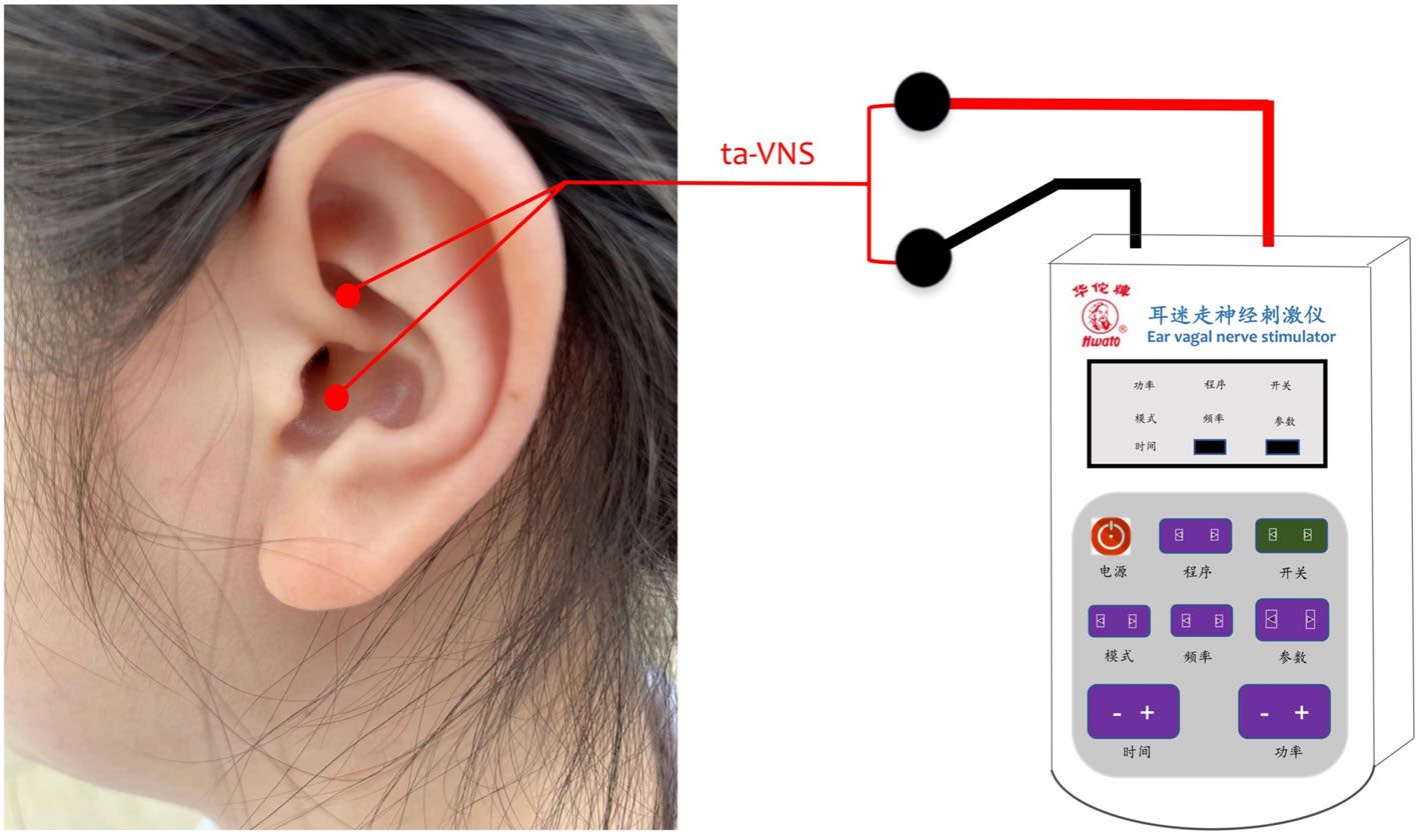
The locations for ta-VNS on the auricular surface. ta-VNS: transcutaneous auricular vagal nerve stimulation.

Specifically, the auricular concha area is the only place on the body surface where the VN sends its peripheral branch, called the auricular branch of the vagus nerve (ABVN)^49^. The ABVN forms a cutaneous receptive field in the pinna of the ear that is highly sensitive to external stimuli. The locations for the application of ta-VNS on the auricular surface are shown in Fig. 1. Evidence for the effects of ta-VNS on brainstem neuronal responses in healthy subjects has been confirmed by fMRI data demonstrating that ta-VNS of the cymba conchae projects to the nucleus tractus solitaries, which is a primary relay station for vagal afferent signals, and its main dopaminergic-downstream targets, the dorsal raphe nucleus, the substantia nigra, the subthalamic nucleus, and a region adjacent to the red nucleus^50^. It is precisely because of this direct anatomical pathway between the ABVN and the brainstem that makes it possible for ta-VNS to regulate bodily functions. Electricity has been an important tool in clinicians' treatments over the past 2000 years, and using electrical stimulation has become one of the optimal way to perform non-invasive or minimally invasive stimulation of the outer ear^51,52^. Although still lacking FDA approval, current evidence suggests several advantages of ta-VNS, including beneficial effects comparable to implantable VNS, simple operation, greater accessibility, and milder side-effects^53–55^^.^

## Hypothesis

Because ta-VNS is as effective as conventional VNS, we hypothesize that parasympathetic activity is increased by ta-VNS so as to suppress sympathetic overactivity and restore sympatho-vagal balance. Our hypothesis assumes that the severity of PCOS and its associated complications can be attenuated by "endogenous systemic braking", which will be naturally provided by the parasympathetic part of the ANS.

## Potential pathways and action mechanisms by which ta-VNS has Therapeutic effects on PCOS

### Energy metabolism regulation via bidirectional vagal signaling

Although obesity is not necessary for the PCOS phenotype, most women with PCOS are overweight or obese. Obesity worsens IR and compensatory hyperinsulinemia (HI), which in turn promotes adipogenesis and reduces lipolysis. In addition, obesity increases the sensitivity of thecal cells to luteinizing hormone stimulation, leading to amplified ovarian androgen production^4^. Furthermore, obesity is often accompanied by systemic or tissue-specific chronic inflammation and oxidative stress, which is also true in the ovary, thus impairing oocyte development and maturation and reducing female fertility^56^. Generally, weight loss is the first step in the management of PCOS^57^, and various weight loss strategies like modified diet, regular exercise, behavioral changes, and even bariatric surgery in cases of severe obesity have been proposed. However, PCOS patients often fail to maintain these interventions and have high drop-out rates, thus decreasing the long-term effects of the interventions. The optimal methods for improving sustainability to achieve the recommended weight loss goal need further research^57^.

In particular, the VN establishes a bidirectional communication between the gastrointestinal tract and the CNS and thus plays an important role in regulating energy metabolism. There is evidence that disrupted vagal signaling is associated with the development of diet-induced weight gain, and targeting the VN with neuromodulation provides a novel way to treat obesity^58^. Previously, several small clinical studies performed in patients with depression or epilepsy observed that VNS might have a significant effect on weight loss^59–61^. Subsequent experiments also showed that chronic VNS can effectively reduce food intake and decrease body weight by increasing brain satiety signals or by delaying gastric emptying mediated via the vagal afferents^62–66^. In addition, restoring or augmenting VN activity can attenuate obesity-associated conditions via inflammatory reflexes^67^. Recently, implanted VNS for obesity has been approved by the FDA^68^. As a non-invasive alternative approach to VNS, ta-VNS is now being investigated to evaluate its effect on weight loss. Li et al. observed that auricular VNS showed remarkable effects on reducing body weight and reducing visceral fat in obese rats^69^. Wang and Yu et al. reported that ta-VNS significantly ameliorates weight gain without changing food intake in Zucker diabetic fatty rats^70–72^. The mechanism might be that ta-VNS can accelerate energy expenditure by inhibiting hypothalamic P2Y1R expression, which is responsible for intracellular triglyceride accumulation in adipocytes mediated by extracellular ATP^73^. Furthermore, the beneficial effects of ta-VNS on body weight and metabolic parameters were shown to produce lasting changes in brain networks^74^. Therefore, it is suggested that ta-VNS might be an alternative therapy for obesity in women with PCOS.

### Reversing insulin resistance via antidiabetic effect

IR is seen in 50% to 70% of women with PCOS^75^ and causes increased secretion of insulin in pancreatic β-islet cells and leads to compensatory HI. IR/HI stimulates increased production of androgens from ovarian theca cells^76^ and inhibits the production of sex hormone binding globulin in the liver, resulting in elevated free testosterone levels in the circulation^77^. IR and HA are considered to be the chief cause of PCOS^22^. It is likely that the presence of IR in PCOS contributes to abnormal glucose homeostasis that further develops into T2DM, and these alterations strongly correlate with the degree of adiposity. On the other hand, the increased androgen secretion associated with PCOS also plays a role in the development of prediabetes and T2DM by exacerbating IR^78^ or by stimulating low-grade inflammation^79^. Clinical evidence has suggested that administration of insulin-sensitizing agents, such as metformin and inositols, along with lifestyle modification can improve the endocrine and metabolic conditions in women with PCOS^80^. However, these agents are still controversial and are associated with some adverse effects such as diarrhea and stomachache^81^. Studies on how to prevent T2DM in women with PCOS are lacking.

The VN plays a necessary role in maintaining glucose homeostasis. The vagal afferent transmits nutrient-dependent signals generated from the upper small intestine to the liver and activates the vagal efferent system at the level of the dorsal vagal complex, thereby regulating hepatic glucose production^82^. Furthermore, the VN is involved in mediating early-phase insulin release as well as optimizing postprandial insulin release from the pancreas^83^. Numerous studies have suggested that ANS imbalance characterized by sympathetic activation or NV inactivity is associated with abnormal glucose metabolism^84^. For example, Poon et al*.* observed that high homeostasis model assessment index for insulin resistance is associated with lower high frequency spectral component, which is an indicator of vagal activity measured during daily activities^85^. The research by Saito et al*.* yielded similar results^86^. Chen et al*.* reported that muscle sympathetic nerve activity burst frequency is inversely correlated with liver insulin sensitivity in non-diabetic obese men^87^. Licht et al. found that high respiratory sinus arrhythmia (which reflects high PNS activity) is negatively associated with glucose levels^88^. Carnethon et al. indicated that lower heart rate variability, which has been used to non-invasively assess efferent vagal pathway activity, is independently associated with the risk of developing T2DM in healthy adults^89^. Given the above association between the VN and glucose metabolism, some studies have provided evidence that targeting vagal signaling to affect glucose metabolism may have therapeutic potential for reversing metabolic disorders. Chunchai et al. and Samniang et al. reported that VNS for 12 weeks significantly improved both peripheral and brain insulin sensitivity in obese IR rats and pigs^90,91^. Huang et al. reported that ta-VNS significantly reduced fasting plasma glucose, 2-h fasting plasma glucose, and glycosylated hemoglobin compared with the no-treatment control group and concluded that ta-VNS is a promising, simple, and cost-effective treatment for impaired glucose tolerance/pre-diabetes with only mild side-effects^92^. Payne et al. and Meyers et al. further suggested that selective efferent VNS might be an effective therapy for treating T2DM^93,94^. As for the therapeutic mechanism, Deng et al. proposed that VNS may prevent IR by activating the α7nACh receptor to attenuate endoplasmic reticulum stress-induced inflammation, thus ameliorating hepatic IR in Kupffer cell^95^. Li et al. demonstrated that long-term ta-VNS treatment may prevent the progression of hyperglycemia possibly through up-regulating insulin receptor expression in various tissues^96^. Wang et al. also suggested that ta-VNS can regulate glucose metabolism by triggering the rhythmic secretion of melatonin, which plays a protective role in T2DM^70^. In addition, Yin et al. reported that VNS reduces blood glucose in diabetic rats by enhancing the release of glucagon-like peptide-1, which can be relayed by vagal afferent neurons to the brain to participate in satiation and glucoregulatory responses^97^. Therefore, we propose the use of ta-VNS to modulate IR in the management of PCOS.

### Activation of anti-inflammatory pathways

Kelly et al*.* proposed for the first time that chronic low-grade inflammation is a novel mechanism contributing to coronary heart disease and T2DM in women with PCOS^98^. Since that work, inflammation has increasingly been recognized as a key contributor to the pathogenesis of PCOS. The chronic low-level inflammation associated with PCOS generally does not show any obvious symptoms such as local redness or fever, but can result in the secretion of inflammatory factors, mainly characterized by elevated concentrations of C-reactive protein (CRP), tumor necrosis factor, interleukin 18 (IL-18), IL-6, white blood cell count, monocyte chemoattractant protein-1, macrophage inflammatory protein-1α, etc.^99^. In addition, all key features of PCOS, such as obesity, IR/HI, and HA aggravate the inflammatory state by promoting the oversecretion of proinflammatory factors^100^. There is also evidence that the uterine hyperinflammatory state in PCOS may induce major pregnancy complications ranging from recurrent miscarriage to placental dysfunction^101^. Therefore, activation of the anti-inflammatory pathways might provide new therapeutic targets for treating PCOS.

Since the late 1990s, the VN has been thought to be a core part of an anti-inflammatory regulatory pathway, which is a part of the inflammatory reflex consisting of both an afferent sensory and efferent effector arm^102^. The anti-inflammatory function of the VN might be mediated by several pathways^103^. The first is the anti-inflammatory hypothalamic–pituitary–adrenal axis (HPAA) pathway. In the HPAA, inflammatory signals are conveyed to the nucleus tractus solitaries via IL-1β receptors in vagal afferents, which activates the A2 noradrenergic group neurons and then sends information to the parvo-cellular region of the paraventricular nucleus of the hypothalamus surrounding corticotrophin releasing factor–containing neurons. These neurons then activate the release of adrenocorticotrophic hormones, which ultimately stimulate the release of glucocorticoids to exert anti-inflammatory effects. The second is the cholinergic anti-inflammatory pathway (ChAP). This pathway is initially activated through afferent VN stimulation, and the signal is then sent to the brain and processed in a muscarinic acetylcholine receptor-dependent manner, and the integrated anti-inflammatory signal is subsequently transmitted via VN efferent fibers to enteric neurons resulting in the release of acetylcholine (Ach), which activates the α7 nicotinic receptors in macrophages causing down-regulation of pro-inflammatory cytokines. The third is the splenic sympathetic anti-inflammatory pathway (SSAP). The inflammatory information is conveyed to the spleen through VN stimulation, and this results in the release of norepinephrine from splenic nerve terminals. Norepinephrine activates β2 adrenergic receptors in specialized T-lymphocytes and promotes the synthesis and expression of Ach. Ach binds to the α7 nicotinic receptors in macrophages, which inhibits the release of pro-inflammatory cytokines. Currently, several preliminary studies have shown a promising effect of the application of VNS as an anti-inflammatory treatment in a range of inflammatory diseases such as sepsis, obesity, CVD, lung injury, diabetes, rheumatoid arthritis, and inflammation-related pain^47^. The development of ta-VNS, which does not need an implanted electrode and neurostimulator, is thus of therapeutic interest. Salama et al*.* showed that ta-VNS attenuates the acute postsurgical inflammatory response by reducing serum CRP, IL6, and IL10 in patients undergoing lobectomy^104^. Animals experiments have also suggested that ta-VNS can suppress inflammatory responses via the α7nAChR-mediated ChAP^105,106^. Therefore, it stands to reason that a sustained increase in vagal nerve activity through ta-VNS treatment may result in a strong anti-inflammatory response by activating the HPAA, ChAP, or SSAP and thus relieving the inflammatory state associated with PCOS.

### Restoring homeostasis of the gut-microbiota-brain axis

In the past two decades, the relationship between the gut microbiota (GM), also called the "second genome" in the human body, and metabolic disorders has become a research hotspot and has provided new insights into the pathogenesis of PCOS^107^. Increasing studies have investigated the potential role of GM dysregulation in the occurrence of PCOS. These studies have reported that compared with healthy controls, women with PCOS have reduced GM diversity and altered barrier function^108,109^, and dysbiotic GM in PCOS is not only associated with metabolic disorders such as IR and obesity^110–112^, but also with reproductive defects such as increased androgen concentrations^113^ and decreased estradiol levels^108^. Zhao et al. concluded in a recent literature review that the GM may promote the development of PCOS through several possible mechanisms, including energy absorption, short-chain fatty acids, lipopolysaccharides, choline and bile acids, intestinal permeability, and the brain-gut axis^107^. Therefore, research targeting the modulation of the GM as a novel therapy for the treatment of PCOS has aroused much interest.

The brain-gut axis is a bidirectional communication system between the brain and the GM through multiple pathways, including the VN, the immune system, neuroendocrine pathways, and bacteria-derived metabolites^114^, of which the afferent vagal pathway is suggested to be the most important^115^. The VN can sense the bacterial compounds or metabolites secreted from the GM through its afferent projections and can transmit this gut information to the CNS where it is integrated and adaptive or inappropriate responses are generated, the latter of which can perpetuate pathological conditions in the digestive tract or favor neurodegenerative diseases^116,117^. Therefore, focusing on the VN might help restore the homeostasis of the microbiota-gut-brain axis to treat these diseases. Compelling evidence have shown that VNS can ameliorate gut dysbiosis-induced pro-inflammatory effects, and VNS may rescue decreased gut mucosal integrity by upregulating enteric glial cells, reducing systemic proinflammatory cytokines, promoting recovery as well as modulating immune functions^118,119^. With regard to the effect of VNS/ta-VNS on the GM profile, there is only the paper by Haney et al. and although their results were neutral, the authors concluded that VNS remains a promising experimental and therapeutic modality for manipulating GM communities^120^. Here, we hypothesize that VNS/ta-VNS can play a positive role in the treatment of PCOS by maintaining the GM homeostasis, and this is worth studying in the future.

### Restoring the sympatho-vagal balance to improve CVD outcomes

CVD is one of the important long-term complications in PCOS patients and is a general clustering of cardiac risk factors. Multiple studies have indicated the relationship between PCOS and an increased risk of CVD^121^. Hudecova et al. found that middle-aged patients with PCOS showed more pronounced endothelial dysfunction in comparison to their age-matched controls^122^. Another 21-year follow-up study indicated that women with PCOS had a higher prevalence of hypertension and increased triglyceride levels during their postmenopausal period than controls^123^. Mani et al. reported a high incidence and age-group-specific prevalence of T2DM, myocardial infarction, and angina among women with PCOS, and over a quarter of them had myocardial infarction or angina when they were older than 65 years^124^. The underlying links between PCOS and CVD are complex and involve various interacting cardiovascular and metabolic factors. These pathophysiological processes include visceral obesity, IR, impaired glucose and lipid homeostasis, HA, and chronic low-grade inflammatory status, all of which appear to responsible for making women with PCOS more prone to developing CVD^125,126^. Consequently, all women with PCOS should be assessed for cardiovascular risk factors. Lifestyle management is recommended as the first-line therapy to prevent primary CVD, and insulin sensitizers, cholesterol-lowering drugs, and other drugs should be administered if dyslipidemia or other risk factors persist^127^.

The ANS plays a pivotal role in the onset and progression of CVD, including hypertension, arrhythmias, coronary artery disease, and heart failure^128^. On the one hand, an increase in cardiovascular sympathetic modulation is associated with poor clinical outcomes and serious complications^129^, while on the other hand the cardiac parasympathetic system appears to be protective against CVD and related mortality both in normal subjects and PCOS patients^130^. Thus, both increased sympathetic and decreased vagal activity may predict abnormal cardiovascular parameters. A growing number of studies have attempted to restore sympatho-vagal balance for the treatment of CVD, and current therapies mainly focus on reducing sympathetic overactivity. However, the appearance of drug resistance and the invasive nature of some surgical procedures have become major challenges to successful treatment. In contrast, targeted stimulation of the parasympathetic branch of the ANS through VNS has shown remarkable effects as an alternative therapeutic method for treating CVD. In the study by Huang et al. the results showed that ta-VNS significantly decreased systolic blood pressure over time compared to sham ta-VNS^92^. Sclocco et al*.* found that mid-intensity respiratory-gated auricular vagal afferent nerve stimulation (RAVANS) could increase the cardiovagal tone and reduce the sympathetic tone during a paced breathing task, which suggested that RAVANS could be used as a non-invasive and inexpensive therapeutic intervention for hypertension^131^. In a prospective study, 24 patients with diastolic dysfunction and preserved left ventricular ejection fraction received two separate 1 h sessions, at least 1 day apart, of active low-level transcutaneous vagus nerve stimulation at the tragus (LLTS) or sham stimulation, and active LLTS treatment acutely improved left ventricular function and favorably altered the heart rate variability frequency domain components^132^. Moreover, the antiarrhythmic properties of ta-VNS on atrial fibrillation were also reported in human studies^133,134^. Given the impressive results of ta-VNS in the treatment of CVD, interventions against the cardiovascular complications of PCOS with non-invasive stimulation approaches such as ta-VNS may lead to a significant improvement in patients’ long-term health.

### Effectively modulating mental disorders

Depression is a common disorder that negatively impairs psychological function and reduces quality of life. A large number of studies have shown that PCOS patients suffers from increased incidence of psychological distress, such as depression^14,135–137^. A recent meta-analysis covering more than 3000 subjects from 10 different countries reported that women with PCOS have twice the prevalence of depression compared to controls (36% vs. 14%), and women with PCOS showed moderate to severe depressive symptoms with an odds ratio of 4.18 compared to women without PCOS^138^. The clear etiology of PCOS-associated depression has not yet been described. The various pathophysiological mechanisms that contribute to depression include IR, disturbance in the HPA axis, androgen excess, inflammation, and the clinical presentations of obesity, hirsutism, and infertility^139,140^. The growing attention to depression has been recognized, and international guidelines now recommend that all women with PCOS should be screened and managed for depression^141^. Weight loss and dietary changes are important measures to prevent PCOS-associated depression. The treatment of PCOS-induced depression is similar to that for depression resulting from other causes, including lifestyle modifications, cognitive behavioral therapy, and pharmacological agents like oral contraceptive pills, metformin, spironolactone, and other antidepressants^139^.

In the past 20 years, a growing number of studies have suggested VNS as a treatment for depression^142–144^. In 2005, VNS was acknowledged by the FDA as an alternative treatment for difficult-to-treat depression in patients more than 18 years old who do not respond to four or more antidepressant treatment protocols^145^. In 2016, with the further development of medical research, the Canadian Network for Mood and Anxiety Treatments recommended VNS as the third-line therapy for drug-resistant depression^146^. Data showed that VNS treatment could improve depression scores by about 25–35%^147^. However, psychiatrists generally do not consider surgical therapy to treat mental illness, even when such therapy is minimally invasive and reversible. Recently, other non-invasive methods, especially ta-VNS, have shown strong therapeutic potential. Numerous animal studies in rat models of stress have suggested a promising role for ta-VNS in alleviating depressive disorder compared to sham ta-VNS^96,148–150^. A recent meta-analysis including four clinical studies with 222 individuals preliminarily demonstrated that ta-VNS could effectively decrease 24-item Hamilton Depression Rating Scale scores, Self-Rating Depression Scale scores, Beck Depression Inventory scores, and Self-Rating Anxiety Scale scores and concluded that ta-VNS was an effective and safe therapy for major depressive disorder^151^. With respect to the potential mechanisms for ta-VNS in the treatment of depression, several hypotheses were put forward, including directly and indirectly modulating the activity and connectivity of key brain regions involved in the pathophysiology of depression, inhibiting neuro-inflammatory sensitization, modulating hippocampal neurogenesis, and regulating the GM-brain-gut axis^152^. Thus, we hypothesize that direct stimulation of the afferent nerve fibers of the ear through ta-VNS produces a similar effect as classic VNS in order to treat PCOS patients with depressive symptoms.

## Conclusion and future direction

**Figure 2:**
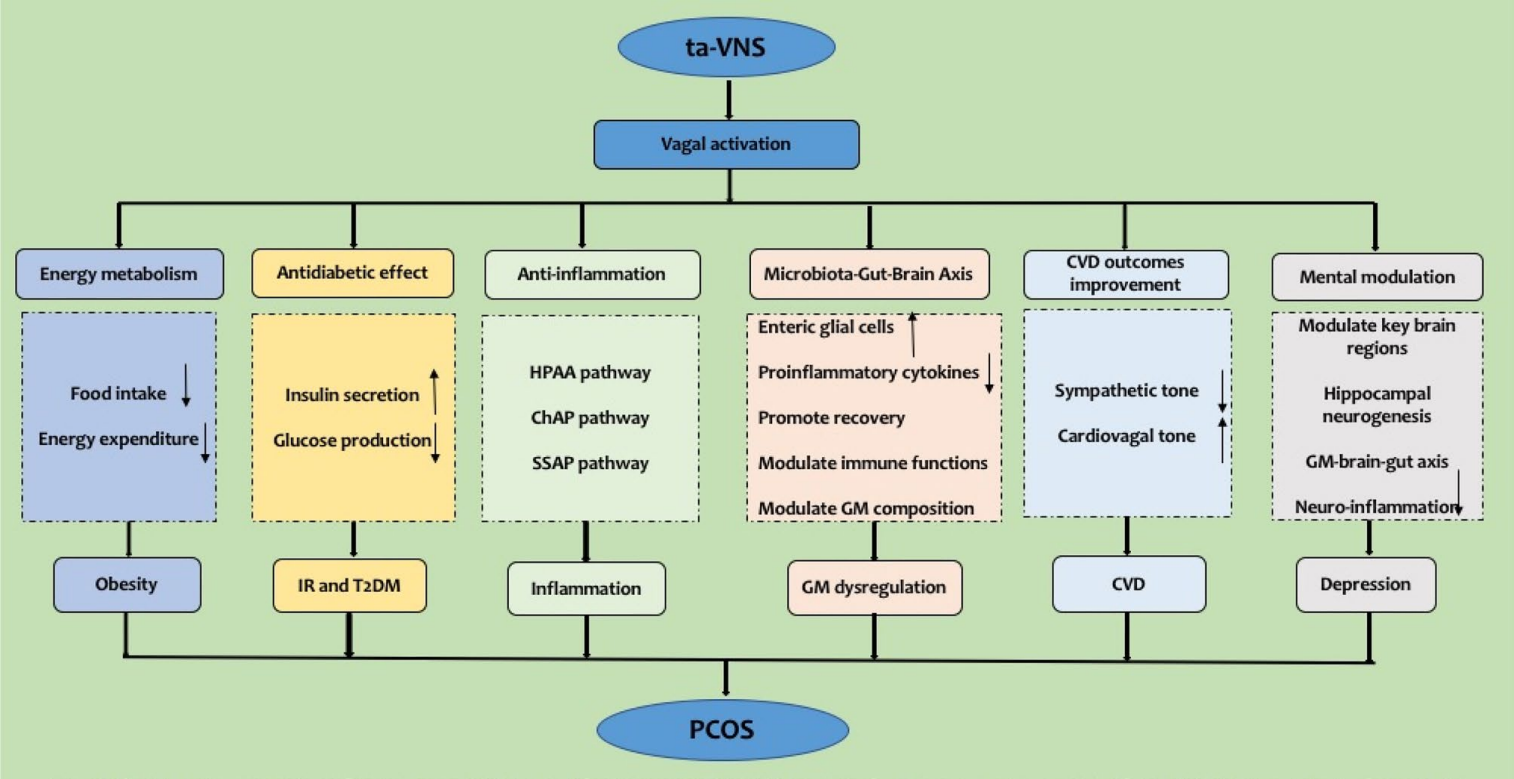
Possible pathways and action mechanisms of ta-VNS in the treatment of PCOS. *ta-VNS* transcutaneous auricular vagal nerve stimulation, *PCOS* Polycystic ovary syndrome, *IR* insulin resistance, *T2DM* type 2 diabetes mellitus, *HPAA* hypothalamic–pituitary–adrenal axis, *ChAP* cholinergic anti-inflammatory pathway, *SSAP* splenic sympathetic anti-inflammatory pathway, *GM* gut microbiota, *CVD* cardiovascular diseases.

ANS dysfunction plays a nonnegligible role in the development of PCOS. The VN belongs to the ANS, and the participation of the VN in regulating ovarian functions has been suggested as an innovative point of departure for studying PCOS. Based on the clinical and experimental findings mentioned above, the enhancement of vagal activity by VNS/ta-VNS may lead to improvement in the various symptoms and complications associated with PCOS, including obesity, IR, T2DM, inflammation, microbiome dysregulation, CVD, and depression. Therefore, we propose a model focusing on ta-VNS that can act on multiple pathways that may treat PCOS (as shown in Table 1 and Fig. 2), including (1) regulating energy metabolism via bidirectional vagal signaling; (2) reversing IR via its antidiabetic effects; (3) activating anti-inflammatory responses through the HPAA, ChAP, and SSAP; (4) restoring homeostasis of the microbiota-gut-brain axis; (5) restoring the sympatho-vagal balance to improve CVD outcomes; and (6) modulating mental disorders. Thus, ta-VNS may be a promising novel therapeutic approach for the treatment of PCOS. The research results for VNS/ta-VNS and for PCOS point to each other; however, there is a lack of relevant research combining the two. Exploring ta-VNS in the management of PCOS is pioneering work, and further studies are needed to dig into the underlying therapeutic mechanisms. Clinical trials with large sample sizes need to be conducted to verify the real effect and long-term safety of ta-VNS in women with PCOS before ta-VNS can be applied in the clinic.

**Table 1:** Potential pathways and action mechanisms for the therapeutic effects of ta-VNS on PCOS.

Potential pathways	Action mechanisms	PCOS-associated complications
Regulation of energy metabolism via bidirectional vagal signaling	Reduce food intake Accelerate energy expenditure	Obesity
Reversing IR via antidiabetic effect	Increase insulin secretion Suppress glucose production	IR and T2DM
Activation of the anti-inflammatory pathways	HPAA pathway ChAP pathway SSAP pathway	Inflammation
Restoring homeostasis of the Microbiota-Gut-Brain Axis	Upregulate enteric glial cells Reduce proinflammatory cytokines Promote recovery Modulate immune functions Modulate GM composition	GM dysregulation
Restoring the sympatho-vagal balance to improve CVD outcomes	Increase the cardiovagal tone Reduce the sympathetic tone	CVD
Effectively modulating mental disorders	Modulate the activity and connectivity of key brain regions Inhibit neuro-inflammatory sensitization Modulate hippocampal neurogenesis Regulate the GM-brain-gut axis	Depression

*ta-VNS* transcutaneous auricular vagal nerve stimulation, *PCOS* Polycystic ovary syndrome, *IR* insulin resistance, *T2DM* type 2 diabetes mellitus, *HPAA* hypothalamic–pituitary–adrenal axis, *ChAP* cholinergic anti-inflammatory pathway, *SSAP* splenic sympathetic anti-inflammatory pathway, *GM* gut microbiota, *CVD* cardiovascular diseases.

## Data Availability

The datasets used in the current study are available from the corresponding author on reasonable request.

## References

[1] Bozdag G, Mumusoglu S, Zengin D (2016). The prevalence and phenotypic features of polycystic ovary syndrome: A systematic review and meta-analysis. Hum. Reprod..

[2] Rotterdam ESHRE/ASRM-Sponsored PCOS Consensus Workshop Group (2004). Revised 2003 consensus on diagnostic criteria and long-term health risks related to polycystic ovary syndrome. Fertil. Steril..

[3] Rotterdam ESHRE/ASRM-Sponsored PCOS consensus workshop group (2004). Revised 2003 consensus on diagnostic criteria and long-term health risks related to polycystic ovary syndrome (PCOS). Hum. Reprod..

[4] Glueck CJ, Goldenberg N (2019). Characteristics of obesity in polycystic ovary syndrome: Etiology, treatment, and genetics. Metabolism.

[5] Hudecova M, Holte J, Olovsson M (2011). Diabetes and impaired glucose tolerance in patients with polycystic ovary syndrome–a long term follow-up. Hum. Reprod..

[6] Celik C, Tasdemir N, Abali R (2014). Progression to impaired glucose tolerance or type 2 diabetes mellitus in polycystic ovary syndrome: A controlled follow-up study. Fertil. Steril..

[7] Kakoly NS, Khomami MB, Joham AE (2018). Ethnicity, obesity and the prevalence of impaired glucose tolerance and type 2 diabetes in PCOS: A systematic review and meta-regression. Hum. Reprod. Update.

[8] Ollila MM, West S, Keinänen-Kiukaanniemi S (2017). Overweight and obese but not normal weight women with PCOS are at increased risk of Type 2 diabetes mellitus-a prospective, population-based cohort study. Hum. Reprod..

[9] Wekker V, van Dammen L, Koning A (2020). Long-term cardiometabolic disease risk in women with PCOS: A systematic review and meta-analysis. Hum. Reprod. Update.

[10] Yin X, Ji Y, Chan CLW (2021). The mental health of women with polycystic ovary syndrome: A systematic review and meta-analysis. Arch. Womens Ment. Health.

[11] Ahmadi M, Faramarzi M, Basirat Z (2020). Mental and personality disorders in infertile women with polycystic ovary: A case-control study. Afr. Health Sci..

[12] Chaudhari AP, Mazumdar K, Mehta PD (2018). Anxiety, depression, and quality of life in women with polycystic ovarian syndrome. Indian J. Psychol. Med..

[13] Brutocao C, Zaiem F, Alsawas M (2018). Psychiatric disorders in women with polycystic ovary syndrome: A systematic review and meta-analysis. Endocrine.

[14] Damone AL, Joham AE, Loxton D (2019). Depression, anxiety and perceived stress in women with and without PCOS: A community-based study. Psychol. Med..

[15] Karjula S, Morin-Papunen L, Auvinen J (2017). Psychological distress is more prevalent in fertile age and premenopausal women With PCOS symptoms: 15-year follow-up. J. Clin. Endocrinol. Metab..

[16] Baskind NE, Balen AH (2016). Hypothalamic-pituitary, ovarian and adrenal contributions to polycystic ovary syndrome. Best Pract. Res. Clin. Obstet. Gynaecol..

[17] Walters KA, Gilchrist RB, Ledger WL (2018). New perspectives on the pathogenesis of PCOS: Neuroendocrine origins. Trends Endocrinol. Metab..

[18] Witchel SF, Tena-Sempere M (2013). The Kiss1 system and polycystic ovary syndrome: Lessons from physiology and putative pathophysiologic implications. Fertil. Steril..

[19] Dumesic DA, Oberfield SE, Stener-Victorin E (2015). Scientific statement on the diagnostic criteria, epidemiology, pathophysiology, and molecular genetics of polycystic ovary syndrome. Endocr. Rev..

[20] Coutinho EA, Kauffman AS (2019). The role of the brain in the pathogenesis and physiology of polycystic ovary syndrome (PCOS). Med. Sci..

[21] Hague WM, Adams J, Rodda C (1990). The prevalence of polycystic ovaries in patients with congenital adrenal hyperplasia and their close relatives. Clin. Endocrinol..

[22] Wang J, Wu D, Guo H (2019). Hyperandrogenemia and insulin resistance: The chief culprit of polycystic ovary syndrome. Life Sci..

[23] Shorakae S, Teede H, de Courten B (2015). The emerging role of chronic low-grade inflammation in the pathophysiology of polycystic ovary syndrome. Semin. Reprod. Med..

[24] Shorakae S, Ranasinha S, Abell S (2018). Inter-related effects of insulin resistance, hyperandrogenism, sympathetic dysfunction and chronic inflammation in PCOS. Clin. Endocrinol..

[25] Dag ZO, Alpua M, Turkel Y (2015). Autonomic dysfunction in patients with polycystic ovary syndrome. Taiwan J. Obstet. Gynecol..

[26] Velusami D, Sivasubramanian S (2018). Sympathovagal imbalance and neurophysiologic cognitive assessment using evoked potentials in polycystic ovary syndrome in young adolescents—a cross-sectional study. J. Basic Clin. Physiol. Pharmacol..

[27] Li W, Chen Y, Xu L (2014). Association of sympathetic nervous system activity with polycystic ovarian syndrome. Clin. Exp. Obstet. Gynecol..

[28] Saranya K, Pal GK, Habeebullah S (2014). Assessment of cardiovascular autonomic function in patients with polycystic ovary syndrome. J. Obstet. Gynaecol. Res..

[29] Gibbons CH (2019). Basics of autonomic nervous system function. Handb. Clin. Neurol..

[30] Butt MF, Albusoda A, Farmer AD (2020). The anatomical basis for transcutaneous auricular vagus nerve stimulation. J. Anat..

[31] Ondicova K, Mravec B (2010). Multilevel interactions between the sympathetic and parasympathetic nervous systems: A minireview. Endocr. Regul..

[32] Farmer AD, Albu-Soda A, Aziz Q (2016). Vagus nerve stimulation in clinical practice. Br. J. Hosp. Med..

[33] Mónica Brauer M, Smith PG (2015). Estrogen and female reproductive tract innervation: Cellular and molecular mechanisms of autonomic neuroplasticity. Auton. Neurosci..

[34] Gerendai I, Tóth IE, Boldogkoi Z (2009). Recent findings on the organization of central nervous system structures involved in the innervation of endocrine glands and other organs; observations obtained by the transneuronal viral double-labeling technique. Endocrine.

[35] Lara HE, Ferruz JL, Luza S (1993). Activation of ovarian sympathetic nerves in polycystic ovary syndrome. Endocrinology.

[36] Barria A, Leyton V, Ojeda SR (1993). Ovarian steroidal response to gonadotropins and beta-adrenergic stimulation is enhanced in polycystic ovary syndrome: Role of sympathetic innervation. Endocrinology.

[37] Lara HE, Dissen GA, Leyton V (2000). An increased intraovarian synthesis of nerve growth factor and its low affinity receptor is a principal component of steroid-induced polycystic ovary in the rat. Endocrinology.

[38] Manni L, Holmäng A, Lundeberg T (2005). Ovarian expression of alpha (1)-and beta (2)-adrenoceptors and p75 neurotrophin receptors in rats with steroid-induced polycystic ovaries. Auton. Neurosci..

[39] Dissen GA, Garcia-Rudaz C, Paredes A (2009). Excessive ovarian production of nerve growth factor facilitates development of cystic ovarian morphology in mice and is a feature of polycystic ovarian syndrome in humans. Endocrinology.

[40] Figueroa F, Mendoza G, Cardozo D (2018). Sympathetic innervation regulates macrophage activity in rats with polycystic ovary. J. Endocrinol..

[41] Hashim ZH, Hamdan FB, Al-Salihi AR (2015). Autonomic dysfunction in women with polycystic ovary syndrome. Iran J. Reprod. Med..

[42] Shorakae S, Abell SK, Hiam DS (2018). High-molecular-weight adiponectin is inversely associated with sympathetic activity in polycystic ovary syndrome. Fertil. Steril..

[43] Sverrisdóttir YB, Mogren T, Kataoka J (2008). Is polycystic ovary syndrome associated with high sympathetic nerve activity and size at birth?. Am. J. Physiol. Endocrinol. Metab..

[44] Lansdown A, Rees DA (2012). The sympathetic nervous system in polycystic ovary syndrome: A novel therapeutic target?. Clin. Endocrinol..

[45] Lanska DJJL (2002). Corning and vagal nerve stimulation for seizures in the 1880s. Neurology.

[46] Wang Y, Zhan G, Cai Z (2021). Vagus nerve stimulation in brain diseases: Therapeutic applications and biological mechanisms. Neurosci. Biobehav. Rev..

[47] Johnson RL, Wilson CG (2018). A review of vagus nerve stimulation as a therapeutic intervention. J. Inflamm. Res..

[48] Giordano F, Zicca A, Barba C (2017). Vagus nerve stimulation: Surgical technique of implantation and revision and related morbidity. Epilepsia.

[49] Wang Yu, Shao-Yuan Li, Dan W (2021). Transcutaneous auricular vagus nerve stimulation: From concept to application. Neurosci. Bull..

[50] Borgmann D, Rigoux L, Kuzmanovic B (2021). Technical note: Modulation of fMRI brainstem responses by transcutaneous vagus nerve stimulation. Neuroimage.

[51] Lewis PM, Thomson RH, Rosenfeld JV (2016). Brain neuromodulation techniques: A review. Neuroscientist.

[52] Kaniusas E, Kampusch S, Tittgemeyer M (2019). Current directions in the auricular vagus nerve stimulation II—an engineering perspective. Front. Neurosci..

[53] Nicholson WC, Kempf MC, Moneyham L (2017). The potential role of vagus-nerve stimulation in the treatment of HIV- associated depression: A review of literature. Neuropsychiatr. Dis. Treat..

[54] Ben-Menachem E, Revesz D, Simon BJ (2015). Surgically implanted and non-invasive vagus nerve stimulation: A review of efficacy, safety and tolerability. Eur. J. Neurol..

[55] Carandina A, Rodrigues GD, Di Francesco P (2021). Effects of transcutaneous auricular vagus nerve stimulation on cardiovascular autonomic control in health and disease. Auton. Neurosci..

[56] Snider AP, Wood JR (2019). Obesity induces ovarian inflammation and reduces oocyte quality. Reproduction.

[57] Jin P, Xie Y (2018). Treatment strategies for women with polycystic ovary syndrome. Gynecol. Endocrinol..

[58] de Lartigue G (2016). Role of the vagus nerve in the development and treatment of diet-induced obesity. J. Physiol..

[59] Bodenlos JS, Kose S, Borckardt JJ (2007). Vagus nerve stimulation acutely alters food craving in adults with depression. Appetite.

[60] Burneo JG, Faught E, Knowlton R (2002). Weight loss associated with vagus nerve stimulation. Neurology.

[61] Pardo JV, Sheikh SA, Kuskowski MA (2007). Weight loss during chronic, cervical vagus nerve stimulation in depressed patients with obesity: An observation. Int. J. Obes..

[62] Bugajski AJ, Gil K, Ziomber A (2007). Effect of long-term vagal stimulation on food intake and body weight during diet induced obesity in rats. J. Physiol. Pharmacol..

[63] Gil K, Bugajski A, Kurnik M (2009). Physiological and morphological effects of long-term vagal stimulation in diet induced obesity in rats. J. Physiol. Pharmacol..

[64] Gil K, Bugajski A, Thor P (2011). Electrical vagus nerve stimulation decreases food consumption and weight gain in rats fed a high-fat diet. J. Physiol. Pharmacol..

[65] Gil K, Bugajski A, Kurnik M (2012). Chronic vagus nerve stimulation reduces body fat, blood cholesterol and triglyceride levels in rats fed a high-fat diet. Folia Med. Cracov..

[66] Dai F, Yin J, Chen JDZ (2020). Effects and mechanisms of vagal nerve stimulation on body weight in diet-induced obese rats. Obes. Surg..

[67] Pavlov VA, Tracey KJ (2012). The vagus nerve and the inflammatory reflex–linking immunity and metabolism. Nat. Rev. Endocrinol..

[68] Pelot NA, Grill WM (2018). Effects of vagal neuromodulation on feeding behavior. Brain Res..

[69] Li H, Zhang JB, Xu C (2016). Effects and mechanisms of auricular vagus nerve stimulation on high-fat-diet-induced obese rats. Nutrition.

[70] Wang S, Zhai X, Li S (2015). Transcutaneous vagus nerve stimulation induces tidal melatonin secretion and has an antidiabetic effect in Zucker fatty rats. PLoS ONE.

[71] Yu Y, He X, Wang Y (2022). Transcutaneous auricular vagal nerve stimulation inhibits limbic-regional P2X7R expression and reverses depressive-like behaviors in Zucker diabetic fatty rats. Neurosci. Lett..

[72] Yu Y, He X, Zhang J (2021). Transcutaneous auricular vagal nerve stimulation inhibits hypothalamic P2Y1R expression and attenuates weight gain without decreasing food intake in Zucker diabetic fatty rats. Sci. Prog..

[73] Burnstock G, Gentile D (2018). The involvement of purinergic signalling in obesity. Purinergic Signal.

[74] Obst MA, Al-Zubaidi A, Heldmann M (2022). Five weeks of intermittent transcutaneous vagus nerve stimulation shape neural networks: A machine learning approach. Brain Imaging Behav..

[75] Bannigida DM, Nayak BS, Vijayaraghavan R (2020). Insulin resistance and oxidative marker in women with PCOS. Arch. Physiol. Biochem..

[76] Garg D, Tal R (2016). Inositol treatment and ART outcomes in women with PCOS. Int. J. Endocrinol..

[77] Sørensen AE, Udesen PB, Wissing ML (2016). MicroRNAs related to androgen metabolism and polycystic ovary syndrome. Chem. Biol. Interact..

[78] Persson S, Elenis E, Turkmen S (2021). Higher risk of type 2 diabetes in women with hyperandrogenic polycystic ovary syndrome. Fertil. Steril..

[79] Duleba AJ, Dokras A (2012). Is PCOS an inflammatory process?. Fertil. Steril..

[80] Pani A, Gironi I, Di Vieste G (2020). From prediabetes to type 2 diabetes mellitus in women with polycystic ovary syndrome: Lifestyle and pharmacological management. Int. J. Endocrinol..

[81] Bahadur A, Arora H, Ravi AK (2021). Comparison of clinical, metabolic and hormonal effects of metformin versus combined therapy of metformin with myoinositol plus D-chiro-inositol in women with polycystic ovary syndrome (PCOS): A randomized controlled trial. Cureus.

[82] Waise TMZ, Dranse HJ, Lam TKT (2018). The metabolic role of vagal afferent innervation. Nat. Rev. Gastroenterol. Hepatol..

[83] Teff KL (2008). Visceral nerves: Vagal and sympathetic innervation. JPEN J. Parenter. Enteral. Nutr..

[84] Lundqvist Martin H, Almby K, Wiklund U (2021). Altered hormonal and autonomic nerve responses to hypo- and hyperglycaemia are found in overweight and insulin-resistant individuals and may contribute to the development of type 2 diabetes. Diabetologia.

[85] Poon AK, Whitsel EA, Heiss G (2020). Insulin resistance and reduced cardiac autonomic function in older adults: The Atherosclerosis Risk in Communities study. BMC Cardiovasc. Disord..

[86] Isao S, Koutatsu M, Eri E (2017). Low heart rate variability and sympathetic dominance modifies the association between insulin resistance and metabolic syndrome—The toon health study. Circ. J..

[87] Chen Daniel LT, Rachael B, Carsten L (2017). Muscle sympathetic nerve activity is associated with liver insulin sensitivity in obese non-diabetic men. Front. Physiol..

[88] Licht CM, Vreeburg SA, van Reedt Dortland AK (2010). Increased sympathetic and decreased parasympathetic activity rather than changes in hypothalamic-pituitary-adrenal axis activity is associated with metabolic abnormalities. J. Clin. Endocrinol. Metab..

[89] Carnethon MR, Golden SH, Folsom AR (2003). Prospective investigation of autonomic nervous system function and the development of type 2 diabetes: The Atherosclerosis Risk In Communities study, 1987–1998. Circulation.

[90] Titikorn C, Bencharunan S, Jirapas S (2016). Vagus nerve stimulation exerts the neuroprotective effects in obese-insulin resistant rats, leading to the improvement of cognitive function. Sci. Rep..

[91] Charles-Henri M, Chloé P, Jean-Louis D (2017). Obesity-associated alterations in glucose metabolism are reversed by chronic ilbateral stimulation of the abdominal vagus nerve. Diabetes.

[92] Huang F, Dong J, Kong J (2014). Effect of transcutaneous auricular vagus nerve stimulation on impaired glucose tolerance: A pilot randomized study. BMC Complement Altern. Med..

[93] Payne SC, Ward G, Fallon JB (2022). Blood glucose modulation and safety of efferent vagus nerve stimulation in a type 2 diabetic rat model. Physiol. Rep..

[94] Meyers EE, Kronemberger A, Lira V (2016). Contrasting effects of afferent and efferent vagal nerve stimulation on insulin secretion and blood glucose regulation. Physiol. Rep..

[95] Jielin D, Meng W, Yankai G (2020). Activation of α7nAChR via vagus nerve prevents obesity-induced insulin resistance via suppressing endoplasmic reticulum stress-induced inflammation in Kupffer cells. Med. Hypotheses..

[96] Li S, Zhai X, Rong P (2014). Therapeutic effect of vagus nerve stimulation on depressive-like behavior, hyperglycemia and insulin receptor expression in Zucker fatty rats. PLoS ONE.

[97] Yin J, Ji F, Gharibani P (2019). Vagal nerve stimulation for glycemic control in a rodent model of type 2 diabetes. Obes. Surg..

[98] Kelly CC, Lyall H, Petrie JR (2001). Low grade chronic inflammation in women with polycystic ovarian syndrome. J. Clin. Endocrinol. Metab..

[99] Rudnicka E, Suchta K, Grymowicz M (2021). Chronic low grade inflammation in pathogenesis of PCOS. Int. J. Mol. Sci..

[100] González F (2015). Nutrient-induced inflammation in polycystic ovary syndrome: Role in the development of metabolic aberration and ovarian dysfunction. Semin. Reprod. Med..

[101] Tersigni C, Vatish M, D'Ippolito S (2020). Abnormal uterine inflammation in obstetric syndromes: Molecular insights into the role of chemokine decoy receptor D6 and inflammasome NLRP3. Mol. Hum. Reprod..

[102] Maier SF, Goehler LE, Fleshner M (1998). The role of the vagus nerve in cytokine-to-brain communication. Ann. N. Y. Acad. Sci..

[103] Bonaz B, Sinniger V, Pellissier S (2016). Anti-inflammatory properties of the vagus nerve: Potential therapeutic implications of vagus nerve stimulation. J. Physiol..

[104] Salama M, Akan A, Mueller MR (2020). Transcutaneous stimulation of auricular branch of the vagus nerve attenuates the acute inflammatory response after lung lobectomy. World J. Surg..

[105] Hong GS, Zillekens A, Schneiker B (2019). Non-invasive transcutaneous auricular vagus nerve stimulation prevents postoperative ileus and endotoxemia in mice. Neurogastroenterol. Motil..

[106] Zhao YX, He W, Jing XH (2012). Transcutaneous auricular vagus nerve stimulation protects endotoxemic rat from lipopolysaccharide-induced inflammation. Evid. Based Complement Alternat. Med..

[107] Zhao X, Jiang Y, Xi H (2020). Exploration of the relationship between gut microbiota and polycystic ovary syndrome (PCOS): A review. Geburtshilfe Frauenheilkd.

[108] Insenser M, Murri M, Del Campo R (2018). Gut microbiota and the polycystic ovary syndrome: Influence of sex, sex hormones, and obesity. J. Clin. Endocrinol. Metab..

[109] Lindheim L, Bashir M, Munzker J (2017). Alterations in gut microbiome composition and barrier function are associated with reproductive and metabolic defects in women with polycystic ovary syndrome (PCOS): A pilot study. PLoS ONE.

[110] Zhang D, Zhang L, Yue F (2015). Serum zonulin is elevated in women with polycystic ovary syndrome and correlates with insulin resistance and severity of anovulation. Eur. J. Endocrinol..

[111] Liang Y, Ming Q, Liang J (2020). Gut microbiota dysbiosis in polycystic ovary syndrome: Association with obesity—a preliminary report. Can. J. Physiol. Pharmacol..

[112] Zhou L, Ni Z, Yu J (2020). Correlation between fecal metabolomics and gut microbiota in obesity and polycystic ovary syndrome. Front. Endocrinol..

[113] Torres PJ, Siakowska M, Banaszewska B (2018). Gut microbial diversity in women with polycystic ovary syndrome correlates with Hyperandrogenism. J. Clin. Endocrinol. Metab..

[114] Bonaz B, Bazin T, Pellissier S (2018). The vagus nerve at the interface of the microbiota-gut-brain axis. Front. Neurosci..

[115] Grewal S, Gupta V (2011). Effect of obesity on autonomic nervous system. Int. J. Curr. Bio Med. Sci..

[116] Eisenstein M (2016). Microbiome: Bacterial broadband. Nature.

[117] Tse JKY (2017). Gut microbiota, nitric oxide, and microglia as prerequisites for neurodegenerative disorders. ACS Chem Neurosci..

[118] Willman J, Willman M, Reddy R (2022). Gut microbiome and neurosurgery: Implications for treatment. Clin. Transl. Discov..

[119] Jakob MO, Murugan S, Klose CSN (2020). Neuro-immune circuits regulate immune responses in tissues and organ homeostasis. Front. Immunol..

[120] Haney MM, Ericsson AC, Lever TE (2018). Effects of intraoperative vagal nerve stimulation on the gastrointestinal microbiome in a mouse model of amyotrophic lateral sclerosis. Comp. Med..

[121] Aziz M, Sidelmann JJ, Faber J (2015). Polycystic ovary syndrome: Cardiovascular risk factors according to specific phenotypes. Acta Obstet. Gynecol. Scand..

[122] Hudecova M, Holte J, Olovsson M (2010). Endothelial function in patients with polycystic ovary syndrome: A long-term follow-up study. Fertil. Steril..

[123] Schmidt J, Landin-Wilhelmsen K, Brännström M (2011). Cardiovascular disease and risk factors in PCOS women of postmenopausal age: A 21-year controlled follow-up study. J. Clin. Endocrinol. Metab..

[124] Mani H, Levy MJ, Davies MJ (2013). Diabetes and cardiovascular events in women with polycystic ovary syndrome: A 20-year retrospective cohort study. Clin. Endocrinol..

[125] Papadakis G, Kandaraki E, Papalou O (2017). Is cardiovascular risk in women with PCOS a real risk? Current insights. Minerva Endocrinol..

[126] Christakou C, Diamanti-Kandarakis E (2013). Structural, biochemical and non-traditional cardiovascular risk markers in PCOS. Curr. Pharm. Des..

[127] Wild RA, Carmina E, Diamanti-Kandarakis E (2010). Assessment of cardiovascular risk and prevention of cardiovascular disease in women with the polycystic ovary syndrome: A consensus statement by the Androgen Excess and Polycystic Ovary Syndrome (AE-PCOS) Society. J. Clin. Endocrinol. Metab..

[128] Hadaya J, Ardell JL (2020). Autonomic modulation for cardiovascular disease. Front Physiol..

[129] Grassi G, Seravalle G, Mancia G (2015). Sympathetic activation in cardiovascular disease: Evidence, clinical impact and therapeutic implications. Eur. J. Clin. Invest..

[130] Thayer JF, Yamamoto SS, Brosschot JF (2010). The relationship of autonomic imbalance, heart rate variability and cardiovascular disease risk factors. Int. J. Cardiol..

[131] Sclocco, R., Garcia, R. G., Gabriel, A. *et al.* Respiratory-gated Auricular Vagal Afferent Nerve Stimulation (RAVANS) effects on autonomic outflow in hypertension. In*Annual International Conference of the IEEE Engineering in Medicine and Biology Society* 3130–3133 (2017). 10.1109/EMBC.2017.8037520 29060561

[132] Tran N, Asad Z, Elkholey K (2019). Autonomic neuromodulation acutely ameliorates left ventricular strain in humans. J. Cardiovasc. Transl. Res..

[133] Stavrakis S, Humphrey MB, Scherlag BJ (2015). Low-level transcutaneous electrical vagus nerve stimulation suppresses atrial fibrillation. J. Am. Coll. Cardiol..

[134] Stavrakis S, Stoner JA, Humphrey MB (2020). TREAT AF (Transcutaneous Electrical Vagus Nerve Stimulation to Suppress Atrial Fibrillation): A randomized clinical trial. JACC Clin. Electrophysiol..

[135] Almis H, Orhon FŞ, Bolu S (2021). Self-concept, depression, and anxiety levels of adolescents with polycystic ovary syndrome. J. Pediatr. Adolesc. Gynecol..

[136] Harnod T, Chen W, Wang JH (2019). Association between depression risk and polycystic ovarian syndrome in young women: A retrospective nationwide population-based cohort study (1998–2013). Hum. Reprod..

[137] Alur-Gupta S, Chemerinski A, Liu C (2019). Body-image distress is increased in women with polycystic ovary syndrome and mediates depression and anxiety. Fertil. Steril..

[138] Cooney LG, Lee I, Sammel MD (2017). High prevalence of moderate and severe depressive and anxiety symptoms in polycystic ovary syndrome: A systematic review and meta-analysis. Hum. Reprod..

[139] Ethirajulu A, Alkasabera A, Onyali CB (2021). Insulin resistance, hyperandrogenism, and its associated symptoms are the precipitating factors for depression in women with polycystic ovarian syndrome. Cureus.

[140] Kolhe JV, Chhipa AS, Butani S (2021). PCOS and depression: Common links and potential targets. Reprod. Sci..

[141] Teede HJ, Misso ML, Costello MF (2018). Recommendations from the international evidence-based guideline for the assessment and management of polycystic ovary syndrome. Fertil. Steril..

[142] George MS, Rush AJ, Marangell LB (2005). A one-year comparison of vagus nerve stimulation with treatment as usualfor treatment-resistant depression. Biol. Psychiatry.

[143] Rush AJ, Marangell LB, Sackeim HA (2005). Vagus nerve stimulation for treatment-resistant depression: A randomized, controlled acute phase trial. Biol. Psychiatry.

[144] Rush AJ, Sackeim HA, Marangell LB (2005). Effects of 12 months of vagus nerve stimulation in treatment-resistant depression: A naturalistic study. Biol. Psychiatry.

[145] Akhtar H, Bukhari F, Nazir M (2016). Therapeutic efficacy of neurostimulation for depression: Techniques, current modalities, and future challenges. Neurosci. Bull..

[146] Kennedy SH, Lam RW, McIntyre RS (2016). Canadian network for mood and anxiety treatments (CANMAT) 2016 clinical guidelines for the management of adults with major depressive disorder: Section 3. Pharmacol. Treat. Can. J. Psychiatry.

[147] Toffa DH, Touma L, El Meskine T (2020). Learnings from 30 years of reported efficacy and safety of vagus nerve stimulation (VNS) for epilepsy treatment: A critical review. Seizure.

[148] Li S, Zhai X, Rong P (2014). Transcutaneous auricular vagus nerve stimulation triggers melatonin secretion and is antidepressive in Zucker diabetic fatty rats. PLoS ONE.

[149] Li S, Wang Y, Gao G (2020). Transcutaneous auricular vagus nerve stimulation at 20 Hz improves depression-like behaviors and down-regulates the hyperactivity of HPA axis in chronic unpredictable mild stress model rats. Front. Neurosci..

[150] Guo X, Zhao Y, Huang F (2020). Effects of transcutaneous auricular vagus nerve stimulation on peripheral and central tumor necrosis factor alpha in rats with depression-chronic somatic pain comorbidity. Neural Plast..

[151] Wu C, Liu P, Fu H (2018). Transcutaneous auricular vagus nerve stimulation in treating major depressive disorder: A systematic review and meta-analysis. Medicine.

[152] Kong J, Fang J, Park J (2018). Treating depression with transcutaneous auricular vagus nerve stimulation: State of the art and future perspectives. Front. Psychiatry.

